# Ability of Group IVB metallocene polyethers containing dienestrol to arrest the growth of selected cancer cell lines

**DOI:** 10.1186/1471-2407-9-358

**Published:** 2009-10-07

**Authors:** Michael R Roner, Charles E Carraher, Kimberly Shahi, Yuki Ashida, Girish Barot

**Affiliations:** 1 University of Texas, Arlington, Department of Biology, Arlington, TX 76010, USA; 2 Florida Atlantic University, Department of Chemistry and Biochemistry, Boca Raton, FL 33431, USA; 3 Florida Center for Environmental Studies, Palm Beach Gardens, FL 33410, USA; 4 Tokushima University, Department of Engineering, Tokushima 770-8506, Japan

## Abstract

**Background:**

Monomeric Group IVB (Ti, Zr and Hf) metallocenes represent a new class of antitumor compounds. There is literature on the general biological activities of some organotin compounds. Unfortunately, there is little information with respect to the molecular level activity of these organotin compounds. We recently started focusing on the anti-cancer activity of organotin polymers that we had made for other purposes and as part of our platinum anti-cancer effort.

**Methods:**

For this study, we synthesized a new series of metallocene-containing compounds coupling the metallocene unit with dienestrol, a synthetic, nonsteroidal estrogen. This is part of our effort to couple known moieties that offer antitumor activity with biologically active units hoping to increase the biological activity of the combination. The materials were confirmed to be polymeric using light scattering photometry and the structural repeat unit was verified employing matrix assisted laser desorption ionization mass spectrometry and infrared spectroscopy results.

**Results:**

The polymers demonstrated the ability to suppress the growth of a series of tumor cell lines originating from breast, colon, prostrate, and lung cancers at concentrations generally lower than those required for inhibition of cell growth by the commonly used antitumor drug cisplatin.

**Conclusion:**

These drugs show great promise in vitro against a number of cancer cell lines and due to their polymeric nature will most likely be less toxic than currently used metal-containing drugs such as cisplatin. These drugs also offer several addition positive aspects. First, the reactants are commercially available so that additional synthetic steps are not needed. Second, synthesis of the polymer is rapid, occurring within about 15 seconds. Third, the interfacial synthetic system is already industrially employed in the synthesis of aromatic nylons and polycarbonates. Thus, the ability to synthesize large amounts of the drugs is straight forward.

## Background

We have synthesized a number of condensation organotin-containing polymers [1-12] and polymeric derivatives of cisplatin [13-17] emphasizing biological activities. Many of these have shown outstanding anticancer and antiviral agents properties as well as the expected antibacterial properties. In some of these efforts we emphasized the use of Lewis bases that themselves offered some biological activity including the known antiviral agent acyclovir and a number of proven antibacterial agents such as norfloxacin, ampicillin, tricarcillin, and ciprofloxacin. Our overall rationale for employing known drugs coupled with metal-containing moieties with characterized biological effects is to create a unique compound that can interact with the target (cancer, bacteria, virus) at several venues. By targeting multiple sites the possibility that the target will become resistant to the treatment is greatly reduced increasing the effectiveness of the treatment.

**Figure 1:**
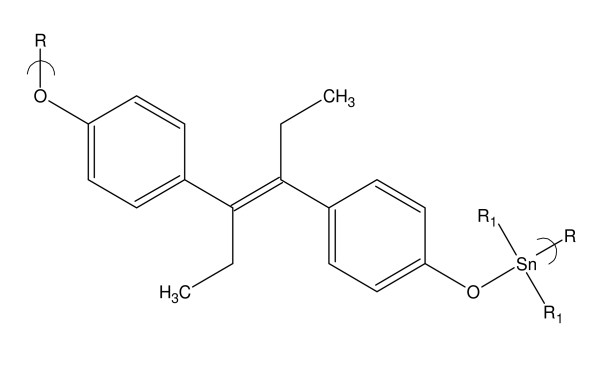
**Repeat unit for the polymer from the reaction of DES and diorganotin dihalides**.

The topic of metallocene polymers has been recently reviewed [18]. We have described the synthesis and ability to arrest the growth of various cells by a number of Group IVB metallocene-containing polymers [1,19-21]. Recently we described the synthesis of organotin polyethers derived from diethylstilbestrol, DES, and the ability of these polymers to arrest the growth of a number of cancer cell lines.^4 ^See Figure 1.

We also recently described the synthesis and ability to arrest the growth of various cell lines for the analogous polyethers derived from Group IVB metallocene dichlorides.

Dienestrol,(4- [4-(hydroxyphenyl)hexa-2,4-dien-3-yl]phenol, is one of the most widely utilized sex hormones. It was initially synthesized in 1939 and initially patented by both Boots and Hoffman-La Roche in 1949 [22,23]. In the popular literature it is often confused with DES, diethylstilbestrol, but it is a distinct hormone with its own chemical and biological properties. It is sold under a variety of tradenames including Farmacyrol, Lipamone, and Retalon-Oral.

**Figure 2:**
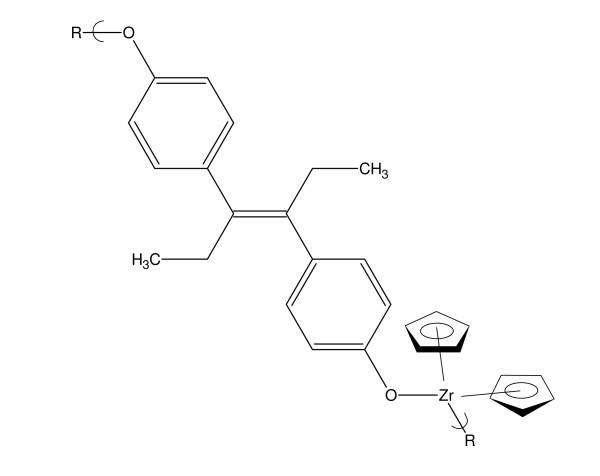
**Repeat unit for the polymer from the reaction of DES and zirconocene dichloride**.

Dienestrol is widely used in hormone therapy, mainly hormone replace therapy or more precisely, estrogen replacement therapy. See Figure 2.

**Figure 3:**
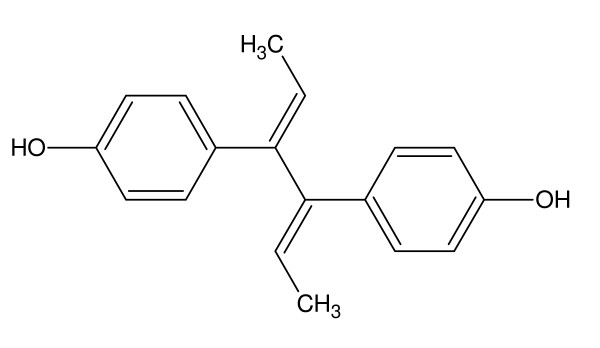
**Structure of dienestrol**.

Interestingly, little work has been done with respect to incorporating dienestrol into polymers either through the double bonds or through the diol moiety. Most of the current efforts with respect to polymers involve the synthesis of dienestrol, and similar drugs, employing covalent molecular imprinting [24-29] Additional work has focused on the controlled release of various steroids, including dienestrol, from various polymeric matrices [30-32]. See Figure 3.

In 1956 a patent described the synthesis of antienzymic polymeric materials from the reaction of various diols including dienestrol from reaction with phosphoric and thiophosphoric acid [33]. It is not clear that any characterization of the product occurred since it was simply one of about 30 diol, diamine, and alcohol-amine-containing Lewis bases included in the patent.

More recently, we described the synthesis of various polyphosphate and polyphosphate esters from the reaction of phosphorous acid chlorides with dienestrol [34].

**Figure 4:**
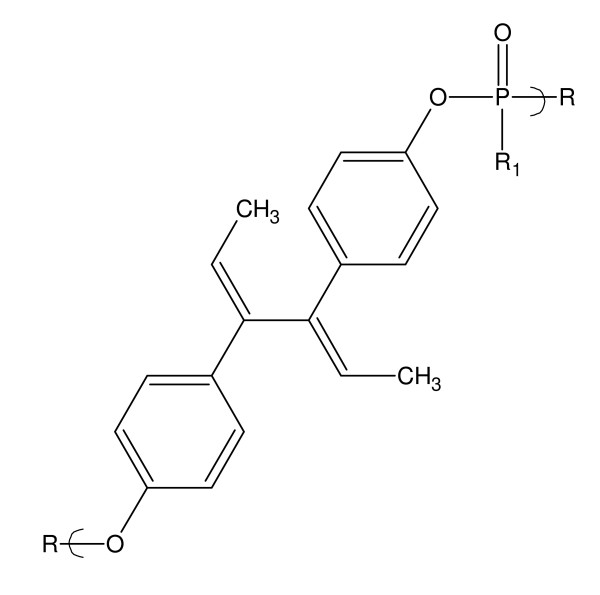
**Repeat unit for the polymer from the reaction of dienestrol and phosphorus acid chlorides**.

About three decades ago we initially synthesized metallocene polyethers including a number of polyethers containing the titanocene, [35-37] zirconocene, [38] and hafnocene units [39]. This effort was recently reviewed [18]. There were a number of reasons for our synthesis of these materials including their use to control high energy radiation, as permanent coloring agents, and as potential anticancer agents [18]. Activity has recently increased in the use of metallocene-containing compounds in the treatment of cancer [40-52]. Much of this effort has focused on the use of titanocene-containing compounds. See Figure 4.

**Figure 5:**
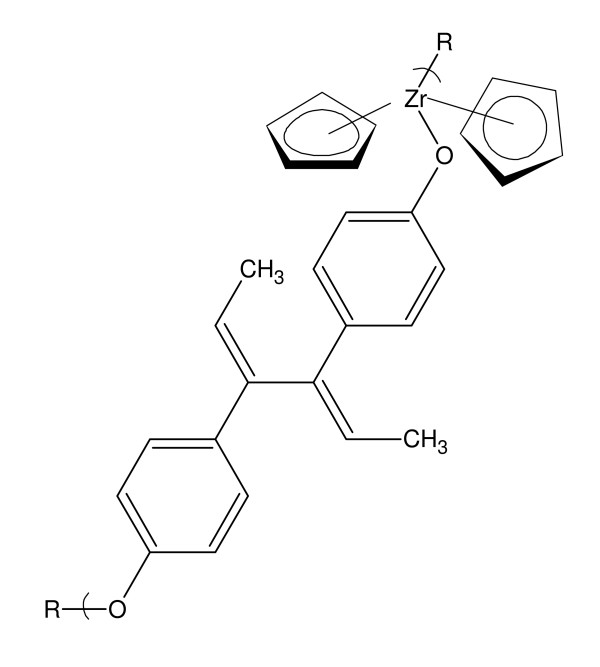
**Repeat unit for the polymer from the reaction of dienestrol and zirconocene dichloride, M = Zr**.

Here we describe the synthesis of Group IVB metallocene polyethers based on the reaction of the Group IVB metallocene dichloride and dienestrol (Figure 5) and preliminary results with respect to their ability to arrest the growth of selected cancer cell lines. The advantages of employing polymeric drugs has been recently reviewed [16,53-55].

## Methods

### Synthesis and Physical Characterization

Polymerization was accomplished employing the classical or aqueous interfacial polycondensation system. Briefly, an aqueous solution (30 mL) containing the dienestrol (0.00300 mol) and sodium hydroxide (0.0060 mole) was transferred to a one quart Kimax emulsifying jar fitted on top of a Waring Blender (model 1120; no load speed of about 18,000 rpm; reactions were carried out at about 25°C). Stirring was begun and a chloroform solution (30 ml) containing the metallocene dihalide (0.00300 mol) was rapidly added (about 3-4 seconds) through a hole in the jar lid using a powder funnel. The resulting solution was blended for 15 seconds. The precipitate was recovered using vacuum filtration and washed several times with deionized water and chloroform to remove unreacted materials and unwanted by-products. The solid was washed onto a glass petri dish and allowed to dry at room temperature.

Chain length was determined employing light scattering photometry using a Brice-Phoenix BP 3000 Universal Light Scattering Photometer. Refractive indicies were obtained using a Bauch & Lomb Model 3-L refractometer. FTIR spectra were obtained employing KBr pellets using a Mattson Instruments galaxy Series 4020 FTIR using 32 scans and an instrumental resolution of 4 cm^-1^.

High resolution electron impact positive ion matrix assisted laser desorption ionization time of flight, HR MALDI-TOF, mass spectrometry was carried out employing a Voyager-DE STR BioSpectrometer, Applied Biosystems, Foster City, CA. The standard settings were used with a linear mode of operation and an accelerating voltage of 25,000 volts; grid voltage 90% and an acquisition mass range of 100 to 2,000 daltons. Two hundred shots were typically taken for each spectrum. Several matrix materials were employed but here only results employing α-cyano-4-hydroxycinnamic acid are included for the hafnocene and zirconocene products and the matrix 2,5-dihydroxybenzoic acid for the titanocene product.

### Biological Characterization

Each of the cell lines listed in Table 1 were obtained from NCI or ATCC and maintained in MEM supplemented with 10% fetal bovine serum at 37°C in a 5% carbon dioxide atmosphere.

**Table 1:** Product yield and molecular weight as a function of metallocene.

Metallocene	% Yield	dn/dc	M_w_	DP
Titanocene	43	-3.5	3.3 × 10^7^	70,000
Zirconocene	42	-1.0	6.7 × 10^6^	14,000
Hafnocene	47	-0.40	2.1 × 10^5^	400

**Note**. While the solubility in DMSO is too low to permit NMR and LS in DMSO it is sufficient to allow for biological testing employing DMSO stock solution.

For testing of the compounds, cells were harvested, counted, and plated into 96-well plates at 1 × 10^4 ^cells per well in MEM-Eagles supplemented with 10% fetal bovine serum, and incubated for 24 hours at 37°C in a 5% carbon dioxide atmosphere. A stock solution of the compound was prepared in DMSO at a known concentration. On day two 100 μL MEM-Eagles supplemented with 10% fetal bovine serum and the indicated drug concentrations was added. Seventy-two hours later the cells were assayed for proliferation using the CellTiter 96^® ^Aqueous One Solution Cell Proliferation Assay by Promega Corporation. The assay is a colorimetric method for determining the number of viable cells in proliferation, cytotoxicity or chemosensitivity assays. The assay solution contains a tetrazolium compound [3-(4,5-dimethylthiazol-2-yl)-5-(3-carboxymethoxyphenyl)-2-(4-sulfophenyl)-2H-tetrazolium, inner salt; MTS^(a)^] and an electron coupling reagent (phenazine ethosulfate; PES). Assays are performed by adding a small amount of the CellTiter 96 Aqueous One Solution Reagent directly to culture wells, incubating for 1-4 hours and then recording absorbance at 490 nm with a 96-well plate reader. The quantity of formazan product as measured by the amount of 490 nm absorbance is directly proportional to the number of living cells in culture.

All cytotoxicity values are calculated against a base-line value for each line that was generated from "mock-treatment" of the normal and tumor cells lines with media supplemented with all diluents used to prepare the chemotherapeutic compounds. For example, if the compounds were dissolved in DMSO and serial dilutions prepared in MEM to treat the cells, then the mock-treated cells were "treated" with the same serial dilutions of DMSO without added chemotherapeutic compound. This was done to ensure that any cytotoxicity observed was due to the activity of the compound and not the diluents. For the studies reported here, the mock-treatment never resulted in a loss of cell viability of more than one percent, demonstrating that the activity observed was not due to cytotoxicity of any of the diluents used, but was due to activity of the tested compounds.

## Results and Discussion

### Synthesis and Structural Characterization

The products were synthesized in average yields in the same range as found for the synthesis of the analogous products except employing DES as the diol in place of dienestrol (Table 1).

The polymers are not soluble in DMF, acetone, and DMF, all solvents that typically dissolved other similar organometallic condensation polymers, and only partially soluble in DMSO. In comparison to the analogous organotin-diethylstilbestrol products, the Group IVB metallocene products have poorer solubilities. This trend is found for other polymers synthesized by us where the metallocene-containing polymers exhibit poor solubility in comparison to the analogous organotin polymers.^17 ^The products are soluble in HMPA. Results appear in Table 1. The products are medium to long chained products.

The products from titanocene and zirconocene are stable in HMPA solution for three months whereas the hanfocene product was stable for 2 months and at month three there was a modest loss in molecular weight from 2.1 × 10^5 ^to 1.7 × 10^5^.

Infrared spectral results are consistent with the proposed repeat unit. Only selected bands will be described here. Here we will concentrate on the IR spectra from the titanocene product. The dienestrol-associated O-H stretch occurs with a maximum at 3415 (all bands given in cm^-1^). It is the most intense band in the spectrum of dienestrol. Dienestrol shows bands due to the aromatic C-H at 3026 and 3005 and aliphatic C-H bands at 2972, 2933, 2910, and 2852. Titanocene dichloride shows a band at 3103 assigned to C-H aromatic stretching. The product shows bands at about 3100, 3027, 3005, 2972, 2910, and 2854. The locations of bands associated with the Ti-O are reported to be about 345 for the symmetric stretch and 420 for the asymmetric stretch. The presence of a new band at 440 is assigned to the Ti-O asymmetric stretch. The 345 band is below the capability of the employed instrument. Other titanocene dichloride, dienestrol, and metallocene polymer-associated bands and assignments are given in Table 2. Thus, IR spectral results are consistent with the proposed structure.

**Table 2:** Vibrational band assignments for titanocene dichloride and the products of Group IVB metallocene dichlorides and dienestrol

Mode	Dien	Cp_2_TiCl_2_	Ti-Polymer	Zr-Polymer	Hf-polymer
OH Stretch	3415				
CH St. Ar	3026,3005	3103	3100,3027, 3091	3100, 3027, 3005	3069, 3029, 3005
CH St. Alip	2972, 2933, 2910, 2852		2972, 2910, 2852	2970, 2912, 2854	2970, 2912, 2866
C = C St. Alip	1641		1651	1650	1650
C = C St. Ar	1603		1608	1604	1606
Phenylene Ske	1504		1500	1506	1501
(Cp) CC St	1440		1425	1423	1426
C-O St	1419		1389	1390	1425
CC Stretch	1364		1330	1331	1333
CH i.p. Bend	1238		1243	1244	1246
Ring Breathing	1166		1171	1171	1171
CH o.p. Bend	1093		1099	1099	1101
C-C St	1062		1040	1040	1040
CH i.p. Bend		1015	1012	1014	1014
CC i.p. Bend	949		956	952	955
CH o.p. Bend	892		902	900	854
CH o.p. Bend		820	829	827	828
CH_2 _Rocking	740		740	740	721
CC o.p. Bend	613		617	619	619

A modified MALDI MS analysis was carried out on the products and reactants [55,56] The most intense ion fragment for dienestrol itself was found at 265 (all ion fragments are given in m/z or m/e = 1 in Daltons) assigned to dienestrol. The next intense was at 212 assigned to dienestrol minus 2 C_2_H_4_.

For brevity, only the results for the titanocene products will be described. Table 3 contains the ion fragments and ion fragment clusters found for the product of titanocene dichloride and dienestrol in the range of 100 to 1,000 daltons.

**Table 3:** Most abundant ion fragment clusters for the product from titanocene dichloride and dienestrol; 100 to 1,000 Da.

m/e	(Proposed) Assignment	m/e	(Proposed) Assignment
129	CpTiO	193	Cp_2_TiO
266	D	289	D, Na
313	U-A	529	U+A, O-Cp
551	U+B-Cp	573	U+B
643	U+Cp_2_TiO	706	U+D

**Figure 6:**
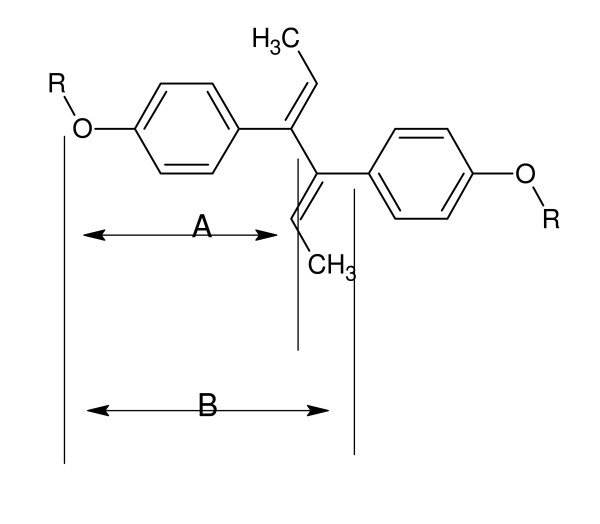
**Fragmentation of dienestrol moiety for MALDI MS fragmentation identification**.

A number of abbreviations are employed to describe the possible ion fragment structures. Some of these are described in Figure 6 below. Additional ones are U = one units; 2 U = 2 units, D = dienestrol, Me = methylene, and Ph = phenylene.

There is some loss of the cyclopentadienyl, Cp, group. This is not unexpected in light of other MS studies that show that metallocene associated Cp groups are especially sensitive to removal from the metallocene moieties [18].

Titanium has five isotopes. This allows isotopic abundance matches to be made. Table 4 contains the isotopic matches for two of these ion fragment clusters. The abundance matches are reasonable and consistent with the presence of one titanium in each ion fragment cluster.

**Table 4:** Isotopic abundance matches for ion fragment clusters centering about 529 and 551 daltons.

		U+A, O-Cp		U+B-Cp	
**m/e**	**% Nat Abu**	**m/e**	**% Rel Abun**	**m/e**	**% Rel Abu**
46	11	527	11	549	12
47	10	528	10	550	11
48	100	529	100	551	100
49	7	530	7	552	8
50	7	531	5	553	7

Table 5 contains the ion fragment clusters found in the range of 10,000 to about 150,000 along with possible assignments. These assignments are to be viewed as suggestive only. This is particularly true because of the ready removal of the Cp group. In other cases such removals occur at only the sites of bond scission so the loss of one or two Cp groups is believed to be typical.

**Table 5:** MALDI MS Results for the mass range of 10,000 to 160,000 Da for the product of titanocene dichloride and dienestrol.

m/e	(Possible) Assignment	m/e	(Possible) Assignment
10823	24U+Cp_2_TiO	11172	25U+B-Cp
11568	26U+A-Cp	13070	30U-Cp_2_TiO
15692	35U+Cp_2_TiO	16750	38U-Cp
17068	39U-Cp_2_Ti	21940	50U-Cp_2_Ti
22219	50U+A-OMe	28310	64U
28863	65U+Cp_2_Ti	32027	72U+Cp_2_Ti
38381	87U-OPh	44590	101U-OPh
48138	109U-OPh	49238	111U-A
49569	112U+O	56009	127U-Cp_2_Ti
56959	129U-O	59220	134U-Cp
67201	152U-Cp+O	84377	191U-2Cp+O
88777	201U-A	94899	214U+D-O
116850	264U+A-Cp	130742	296U-Cp_2_TiO
141309	319U+Cp_2_TiO	142044	321U
154454	349U+A-Cp		

The presence of these high mass ion fragments is consistent with the polymeric nature of the tested material. A number of these ion fragments may be entire chains such as those at 17068, 21940, 22219, 28310, 49238, 56009, 88777, and 142044 consistent with the "soft" nature of MALDI MS.

The results are consistent with other studies where chain scission occurs mainly at the heteroatoms. They are also consistent with the assigned product structure.

### Cell Line Results

The tested cell lines represent a wide range of cancers including breast, lung, colon, and prostate cancer cell lines (Table 6). They also include two "normal" cell lines, the WI-38 human cells which are typically employed as a normal cell line in such studies, and 3T3 cells that are mouse cells that are partially transformed. Both of these cell lines are often employed as examples of healthy cell lines and the results from them are compared to results from the various cancer cell lines giving measures of the ability of the tested drug to differentiate between healthy and cancer cell lines. Of the two, the WI-38 is more widely employed as representative of a healthy cell line for such comparisons. Also included in the test samples is cisplatin, a widely employed anticancer drug, as a comparison to a known anticancer agent.

**Table 6:** Cell line Characteristics and Identification

Strain	NCI/ATCC designation	Species	Tumor orgin	Histological type
3465	PC-3	Human	Prostate	Carcinoma
7233	MDA MB-231	Human	Breast pleural effusion	Adenocarcinoma
1507	HT-29	Human	Colon recto-sigmoid	Adenocarcinoma
7259	MCF-7	Human	Breast pleural effusion	Adenocarcinoma
CCL-75	WI-38	Human	Lung normal embryonic	Fibroblast
CRL-1658	NIH 3T3	Mouse	Continuous cell line of highly contact-inhibited cells	Embryo-fibroblast

Different measures are employed in the evaluation of cell line results. Here we use the two most widely employed- GI_50 _values which is the lowest concentration where growth is inhibited by 50% and the Chemotherapeutic Index, CI_50 _which is a measure of the amount needed to inhibit 50% cell growth, GI_50_, for the normal cell lines, here WI-38 and 3T3 cell lines, divided by the amount needed to inhibit 50% cell growth for one of the cancer cell lines. It is to be noted that different researchers generally emphasize one of these measures over the other with neither measure universally accepted. Thus, results from both of these measures are presented. [see Additional file 1: Table S1] contains the GI_50 _values for the polymers, DES, and cisplatin.

The GI_50 _values for the polymers are significantly lower than for cisplatin with the exception of the WI-38 healthy cells. The GI_50 _values for the metallocene monomers are much higher than for the analogous polymers. They are in the general range of the dienestrol itself. Further, with respect to the polymers the breast cancer cell line without estrogen (MDA MB-231) showed better test results than the breast cancer cell line that is positive for estrogen (MCF-7) perhaps because some of the drug is bound to the estrogen receptors and not available to act within the cell. This is consistent with the finding that DES is effective against estrogen receptor positive (ER+) tumors [57,58].

The second measure is the 50% chemotherapeutic index, CI_50_. When describing the CI some researchers employ the similar terms ED or effective dose in place of the GI value for the cancer cells and LD or lethal dose in place of GI for the healthy cell line, here the WI-38 cell line. Thus, CI_50 _= LD_50_/ED_50_. Larger values are desired since they indicate that a larger concentration is required to inhibit the healthy cells in comparison to the cancer cells or stated in another way, larger values indicate some preference for inhibiting the cancer cells in preference to the normal cells. In general, CI_50 _values larger than 2 are considered significant. CI_50 _values are given in [see Additional file 2: Table S2].

An additional question concerns the influence of simply having the metallocene moiety present in a polymer. [see Additional file 3: Table S3] contains the CI_50 _values normalized against the CI_50 _value for dienestrol. This ratio may offer some measure of the effectiveness of having the two monomer moieties within the polymer. Values greater than one would be consistent with the presence of that moiety present in the polymer having a favorable effect.

## Conclusion

Several features are apparent. First, cisplatin, while a most widely employed anticancer drug, has small CI_50 _values, all 0.02 and lower. Second, the metallocene dichlorides all have low CI_50 _values. Third, dienestrol has two CI_50 _values greater than two. Thus, it shows some significant ability to inhibit the cell growth of the transformed, 3T3, and one cancer drug in comparison to the concentration where the healthy WI-38 cell line is not affected. Third, the metallocene polymers all exhibit some CI_50 _values above 2. The hafnocene and zironocene-dienestrol polymers exhibit high CI_50 _values for all of the tested cancer and transformed cell lines. This is consistent with these particular polymers meriting further study. Further, while much of the recent anticancer effort with respect to metallocene-containing compounds has focused on titanocene-containing compounds, the zirconocene and hafnocene polymers showed somewhat better CI_50 _values in the current study. Thus, future studies might consider not only the titanocene-containing compounds but also the analogous zirconocene and hafnocene compounds for study. Fourth, while the dienestrol exhibited decent CI_50 _values, all of the corresponding polymers exhibited higher CI_50 _values for all of the cell lines. Thus, the ability to arrest cell growth is not only dependent on the presence of the dienestrol but also on the presence of the metallocene moiety.

With respect to the normalization of the CI_50 _values for each cell line against the CI_50 _values for dienestrol itself, no norm has been established for this kind of comparison. Thus, conclusions should be viewed in this light. All of the values for the polymers are greater than two consistent with the presence of having the combination of dienestrol and metallocene in the same polymer being positive with respect to the ability to inhibit cancer and transformed cells in comparison to the healthy WI-38 cell line.

The current drugs also offer several addition positive aspects. First, the reactants are commercially available so that additional synthetic steps are not needed. Second, synthesis of the polymer is rapid, occurring within about 15 seconds. Third, the interfacial synthetic system is already industrially employed in the synthesis of aromatic nylons and polycarbonates. Thus, the ability to synthesize large amounts of the drugs is straight forward.

## Competing interests

The authors declare that they have no competing interests.

## Authors' contributions

YA, GB and CC synthesized a new series of metallocene-containing compounds and preformed the light scattering photometry, the matrix assisted laser desorption ionization mass spectrometry and infrared spectroscopy. KS and MR carried out all the in vitro antitumor activity assays. MR and CC participated in the design of the study and KS performed the statistical analysis. MR and CC conceived of the study, and participated in its design and coordination. All authors read and approved the final manuscript.

## Pre-publication history

The pre-publication history for this paper can be accessed here:

http://www.biomedcentral.com/1471-2407/9/358/prepub

## Supplementary Material

Additional file 1 **Table S1**. GI_50 _concentrations (μg/mL) for metallocene polyethers for tested cell lines ^[*a*]^. [a]The data shown here are the average from three independent experiments, with the standard deviations shown in (). Click here for file

Additional file 2 **Table S2**. CI_50 _concentrations (μg/mL) for metallocene polyethers for tested cell lines ^[*a*]^. [a]The data shown here are the average from three independent experiments, with the standard deviations shown in (). Click here for file

Additional file 3 **Table S3**. CI_50 _concentrations (mg/mL) for the Samples for Each Cell Line Normalized Against Values for Dienestrol. Click here for file
