# Supplementary material for: Ability of Group IVB metallocene polyethers containing dienestrol to arrest the growth of selected cancer cell lines
Source: BMC Cancer. 2009 Oct 7;9:358. doi: 10.1186/1471-2407-9-358 (PMC2765989; doi:10.1186/1471-2407-9-358)
Supplement: Additional file 1 — Table S1. GI50 concentrations (μg/mL) for metallocene polyethers for tested cell lines [a]. [a]The data shown here are the average from three independent experiments, with the standard deviations shown in (). [file 1471-2407-9-358-S1.PDF]

Compound	Structure	Cell line tested					
		WI-38	3T3	PC-3	MDA	HT-29	MCF-7
		GI_{50}					
Dienestrol	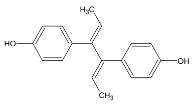	0.25(.2)	0.05(.01)	0.66(.05)	0.11(.02)	0.31(.02)	0.44(.05)
Cp_2Ti	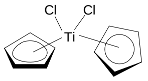	19(1.2)	81(5.1)	29(3.0)	46(3.2)	35(4.6)	112(8.5)
Cp_2Zr	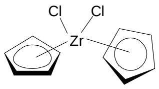	19(1.2)	40(3.2)	39(3.6)	38(3.2)	53(4.6)	122(9.0)
Cp_2Hf	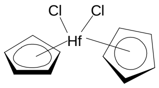	18(1.2)	41(3.7)	36(3.8)	45(3.2)	53(4.6)	94(8.0)
Cp_2Ti/Die	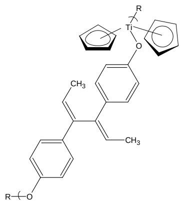	0.91(.3)	0.07(.01)	0.59(.05)	0.08(.01)	0.09(.01)	0.72(.05)
Cp_2Hf/Die	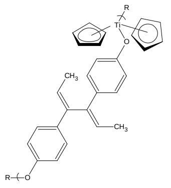	2.5(.5)	0.07(.01)	0.61(.05)	0.16(.02)	0.24(.02)	0.55(.05)
Cp_2Zr/Die	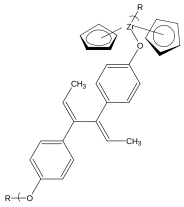	2.0(.5)	0.05(.01)	0.45(.05)	0.10(.01)	0.10(.01)	0.44(.05)
Cisplatin	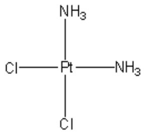	0.05(0.04)	3.00(0.29)	1.00(0.10)	1.00(0.10)	2.00(0.21)	3.00(0.28)
