# Supplementary material for: Ability of Group IVB metallocene polyethers containing dienestrol to arrest the growth of selected cancer cell lines
Source: BMC Cancer. 2009 Oct 7;9:358. doi: 10.1186/1471-2407-9-358 (PMC2765989; doi:10.1186/1471-2407-9-358)
Supplement: Additional file 2 — Table S2. CI50 concentrations (μg/mL) for metallocene polyethers for tested cell lines [a]. [a]The data shown here are the average from three independent experiments, with the standard deviations shown in (). [file 1471-2407-9-358-S2.PDF]

Compound	Structure	Cell line tested				
		WI-38/ 3T3	WI-38/ PC-3	WI-38/ MDA	WI-38/ HT-29	WI-38/ MCF-7
		CI_{50}				
Dienestrol	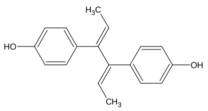	5.0	0.4	2.2	0.8	0.6
Cp_2Ti	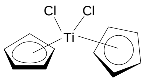	0.2	0.7	0.4	0.4	0.2
Cp_2Zr	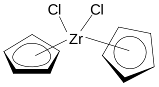	0.5	0.5	0.5	0.4	0.2
Cp_2Hf	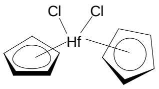	0.5	0.5	0.4	0.3	0.2
Cp_2Ti/Die	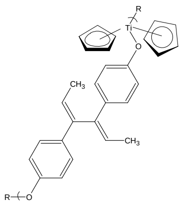	13	1.5	11.0	10.0	1.3
Cp_2Hf/Die	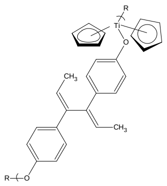	36	4.1	16.0	10.0	4.5
Cp_2Zr/Die	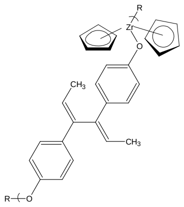	25	3.5	11.5	9.0	3.0
Cisplatin	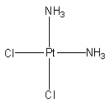	0.02	0.05	0.05	0.03	0.02
