# Supplementary material for: Ability of Group IVB metallocene polyethers containing dienestrol to arrest the growth of selected cancer cell lines
Source: BMC Cancer. 2009 Oct 7;9:358. doi: 10.1186/1471-2407-9-358 (PMC2765989; doi:10.1186/1471-2407-9-358)
Supplement: Additional file 3 — Table S3. CI50 concentrations (mg/mL) for the Samples for Each Cell Line Normalized Against Values for Dienestrol. [file 1471-2407-9-358-S3.PDF]

Compound	Structure	Cell line tested				
		WI-38/ 3T3	WI-38/ PC-3	WI-38/ MDA	WI-38/ HT-29	WI-38/ MCF-7
		CI_{50}				
Dienestrol	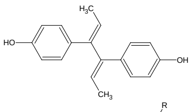	1.0	1.0	1.0	1.0	1.0
Cp_2Ti/Dienestrol	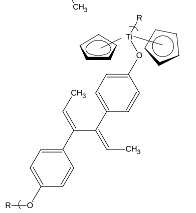	2.6	3.8	5.0	13.0	2.2
Cp_2Hf/Dienestrol	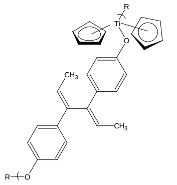	7.2	10.0	7.3	13.0	7.5
Cp_2Zr/Die	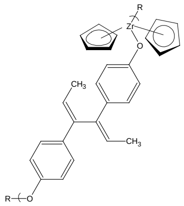	5.0	8.8	5.2	11.3	5.0
